# A retrospective study evaluating the influence of Class III correction appliances on the sagittal pharyngeal airway dimension

**DOI:** 10.1038/s41598-024-57614-w

**Published:** 2024-03-28

**Authors:** Farah Y. Eid, Bassant A. Abbas, Dina A. Elfouly, Ahmed M. Madian

**Affiliations:** Department of Orthodontics, Faculty of Dentistry, Alexandria University, Champolion Street, Azarita, Alexandria, Egypt

**Keywords:** Class III, Facemask, Reversed forsus fatigue resistant device, CS-2000, Sagittal pharyngeal airway dimension, Health care, Medical research

## Abstract

The aim of this study was to compare the effects of Class III correction appliances including the Facemask (FM), and the new non-compliance fixed functional appliances such as the Reversed Forsus Fatigue Resistant Device (FRD), as well as the CS-2000 (CS), on the sagittal pharyngeal airway dimension (SPAD). Pre-treatment and post-treatment lateral cephalograms of 45 patients who underwent Class III appliance treatment, using either FM, Reversed FRD, or CS were collected from the files of treated patients. SPAD changes were evaluated in each group, and comparisons were conducted between the three study groups. Additionally, sagittal and vertical skeletal measurements were conducted. The FM, the Reversed FRD, and the CS, were found to generate a significant increase in the SPAD, with the Reversed FRD contributing to the most significant change at the OPAA (116.80 ± 26.36 mm^2^). All three appliances elicited significant antero-posterior changes in the SNA°, SNB°, and ANB°, also with the greatest intermaxillary change documented with the employment of the Reversed FRD (ANB° = 3.33 ± 0.82°). As for the vertical dimension, the FM, the Reversed FRD, and the CS elicited significant FMA° increases, with the greatest change attributed to the FM (FMA° = 2.32 ± 0.97°). Therefore, the three tested Class III corrective appliances generated significant SPAD, antero-posterior, and vertical changes. However, the Revered FRD showed a superior impact in increasing the SPAD at the OPAA level and in eliciting significant intermaxillary changes.

## Introduction

Class III malocclusions are considered among the most challenging orthodontic anomalies, that are rather arduous to treat^1^. They are known to originate from skeletal and/or dental deformities, or a combination of both^2,3^. Correction and treatment of this type of malocclusion is largely dependent on the growth status of the involved patient, as well as the jaw from which the problem originates^2^.

Maxillary protraction appliances (MPA) have been employed in skeletal Class III correction since a very long time^4^. In a myriad of studies, it has been displayed that they successfully stimulate forward maxillary displacement, and opposingly, reduce the forward mandibular displacement^5–8^. Facemask (FM) therapy is the conventional and the most widely utilized appliance in the phase of growth and development, where a significant orthopaedic effect is produced through maxillary protraction and the strain employed on the circum-maxillary sutures^9,10^. However, since the outcome of removable appliances is dependent on patient compliance to a great extent, recent research has been focused on fixed functional appliances as alternatives to overcome this downside^11,12^.

One of the non-compliance fixed functional appliances proposed for Class III correction is the Reversed Forsus Fatigue Resistant Device (FRD) (3M, Unitek). Initially, the FRD was proposed by William B. Vogt^13^ in 2003, for the treatment of Class II malocclusions. The FRD was subsequently modified by reversing it into the Reversed FRD, to help in the correction of Class III. Successful results have been reported regarding its effectiveness, where a considerable improvement in the intermaxillary sagittal relationship has been noted through the improved forward maxillary growth, as well as the mesial movement of the maxillary dentition^12,14,15^.

Another non-compliance fixed functional appliance for the correction of Class III is the CS-2000 (CS) (Dynaflex, St.Ann, MO, USA)^16^. This appliance is a fixed inter-arch spring-loaded module, with bilateral nickel–titanium (NiTi) closed-coil springs that could be employed in the same manner as Class III elastics^16^. Utilizing the CS-2000 has been found to correct the overjet in Class III cases through forward movement of the maxilla, downward backward movement of the mandible, as well as mesialization of the maxillary teeth^16^.

Pharyngeal size is a fundamental factor affecting the quality of sleep that the patient exhibits^17,18^. Moreover, the size of the nasopharynx has a detrimental effect on the pattern of breathing, whether it is oral or nasal^19^. Augmenting the pharyngeal airway space could be accomplished through medical, surgical, and orthodontic treatments^19^. Despite the fact that the positive impact of maxillary protraction appliances on the pharyngeal space has been highlighted in several investigations^19–21^, there is no concurrence regarding the skeletal and airway changes induced by the various Class III therapies, whether FM with or without rapid maxillary expansion, or chin cup therapy, and the need for high quality studies with a large sample size is always recommended^22^. Additionally, after a thorough review of the existent literature, no study has been reported to compare the influence of different Class III correction appliances on the pharyngeal airway dimension, including the recent non-compliance appliances such as the Reversed FRD and the CS-2000.

Accordingly, the aim of the current study was to compare the effects of three Class III correction appliances including the FM, the Reversed FRD, and the CS on the sagittal pharyngeal airway dimension (SPAD) in skeletal Class III subjects. Moreover, sagittal and vertical skeletal changes accompanying each of the tested appliances were assessed. The null hypothesis was that there is no difference between the three studied appliances regarding their effect on the SPAD.

## Methods

### Patient selection

This retrospective study was approved by the Institutional Review Board of the Faculty of Dentistry, Alexandria University, Alexandria, Egypt (IRB:00010556–IORG:0008839). Manuscript Ethics Committee number 0652-03/2023. Forty-five pre-treatment and post-treatment lateral cephalograms of patients treated at the Orthodontic Department, Faculty of Dentistry, Alexandria University until March 2022, were screened for eligibility by the principal investigator. All the research procedures were performed in accordance with the relevant guidelines and regulations, as stated in the Declaration of Helsinki. Oral assents and written informed consents were obtained from the patients and/or their legal guardian(s) for study participation and for publication of their records and/or identifying information/images in an online open access publication.

The sample size was estimated assuming 80% study power, and 5% alpha error. Yavan et al.^12^ reported mean (SD) change in ANB° = 3.95 (1.11), and 2.36 (1.09) after treatment with facemask and reversed forsus appliances, respectively. Vanlaecken et al.^16^ reported mean (SD) change in ANB° after treatment with CS-2000 appliance = 2.60 (1.70). It is assumed that there is an association between the airway dimensions and ANB° angle; as the ANB° angle increases, the airway dimension is increased^23^. The minimum sample size was calculated to be 15 per group, and the total required sample size = number of groups × number per group = 3 × 15 = 45 patients^24^. The employed software for sample size calculation was G*Power Version 3.1.9.7.

Inclusion criteria for patient selection included an age range from 8 to 11 years (mean age 9.4 ± 0.9 years), skeletal Class III subjects with maxillary deficiency, in addition to an initial ANB angle ranging from − 4° to 0° and a pre-treatment anterior cross bite, that have been treated with either a Facemask, a Reversed FRD, or a CS, and finally, the availability of pre-treatment and post-treatment lateral cephalometric radiographs. In the Reversed FRD and the CS subjects, the appliances were installed on metal bands placed on the corresponding teeth. In all the included subjects, treatment was terminated after reaching a positive overjet. Moreover, the growth status of the chosen sample was dictated from the cervical vertebral maturational index (CVMI), as stated by Hassel and Farman^25^, using lateral cephalograms. Within the selected age range (8–11 years), the CVMI were within 1 and 2. As for the exclusion criteria, they included transverse maxillary deficiency requiring expansion, history of orthodontic treatment, cleft lip and/or palate, craniofacial anomalies or diagnosed syndromes, in addition to history of tonsillectomy or maxillofacial surgery.

The procured lateral cephalograms were taken using a standardized technique with the same machine, where patients stood in natural head position^26,27^, and natural tongue posture with the teeth in centric occlusion. Subjects were instructed to stand still, and not to move their heads nor swallow during exposure. Lateral cephalograms were compared between pre-treatment (T1) and post-treatment (T2) for the analysis of the SPAD, as well as the vertical and sagittal skeletal alterations in the three intervention groups.

### Grouping

**Figure 1:**
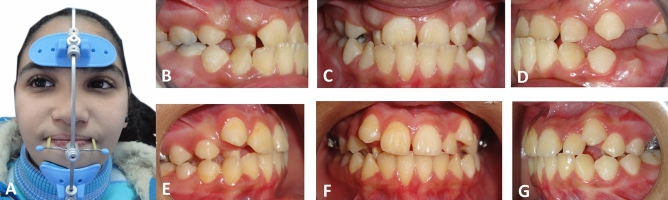
(**A**) Extra-oral view of the employed FM. (**B**, **C**, **D**) Pre-treatment intra-oral views. (**E**, **F**, **G**) Post-treatment intra-oral views, after termination of FM therapy.

**Figure 2:**
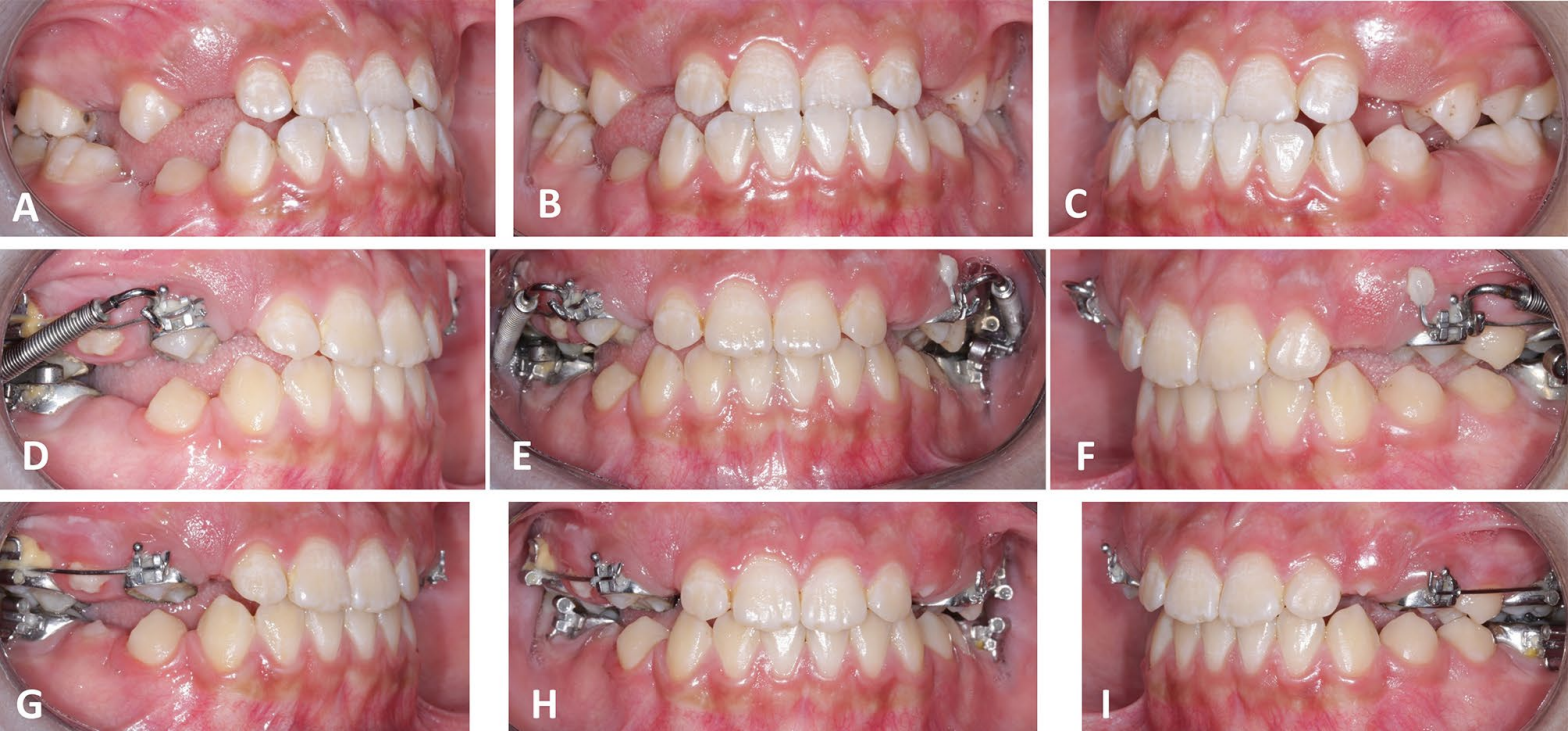
(**A**, **B**, **C**) Pre-treatment intra-oral views prior to using the Reversed FRD appliance. (**D**, **E**, **F**) Progress intra-oral views with the Reversed FRD appliance in place. (**G**, **H**, **I**) Post-treatment intraoral views.

**Figure 3:**
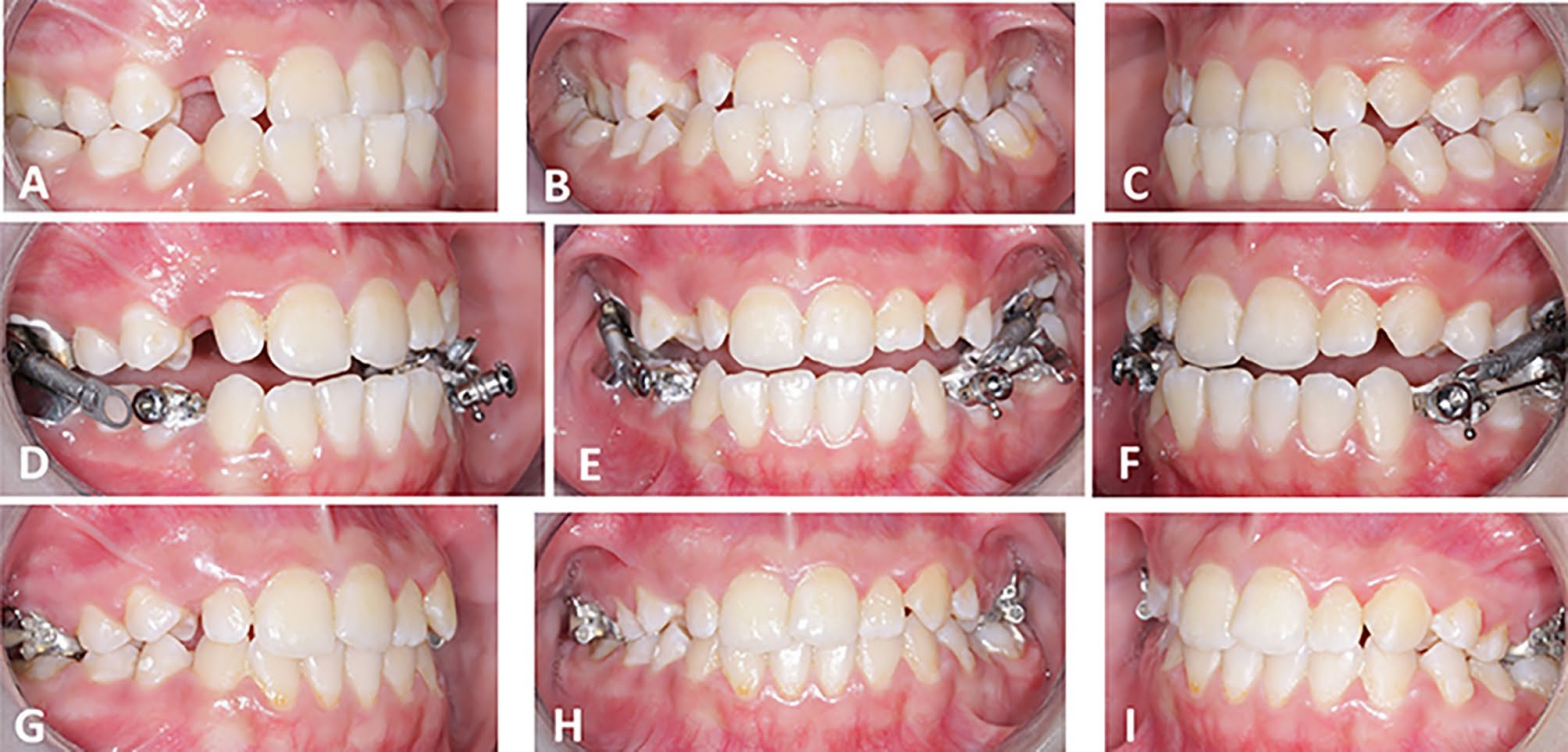
(**A**, **B**, **C**) Pre-treatment intra-oral views prior to employing the CS-2000 appliance. (**D**, **E**, **F**) Progress intra-oral views with the CS-2000 appliance in place. (**G**, **H**, **I**) Post-treatment intraoral views.

The selected lateral cephalometric records were divided into three groups based on the employed Class III corrective appliance; Group A: Facemask therapy (Osstem Orthodontics Inc., South Korea) (Fig. 1), Group B: Reversed Forsus Fatigue Resistant Device (3M, Unitek) (Fig. 2), Group C: CS-2000 (Dynaflex, St. Ann, MO, USA) (Fig. 3).

### Outcome measurements

#### Sagittal pharyngeal airway dimension (area measurement)

**Figure 4:**
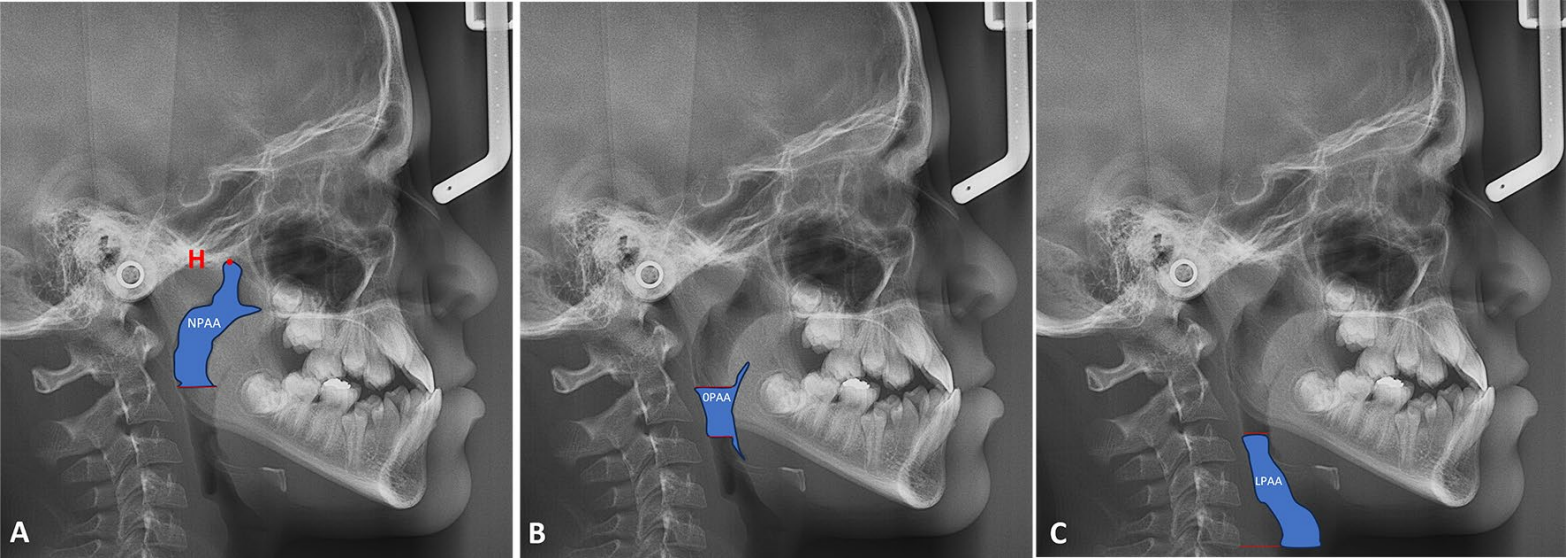
The implemented cephalometric sagittal pharyngeal airway area measurements (H; Harmonium). (**A**) Nasopharyngeal airway area (NPAA). (**B**) Oropharyngeal airway area (OPAA). (**C**) Laryngopharyngeal airway area (LPAA).

**Figure 5:**
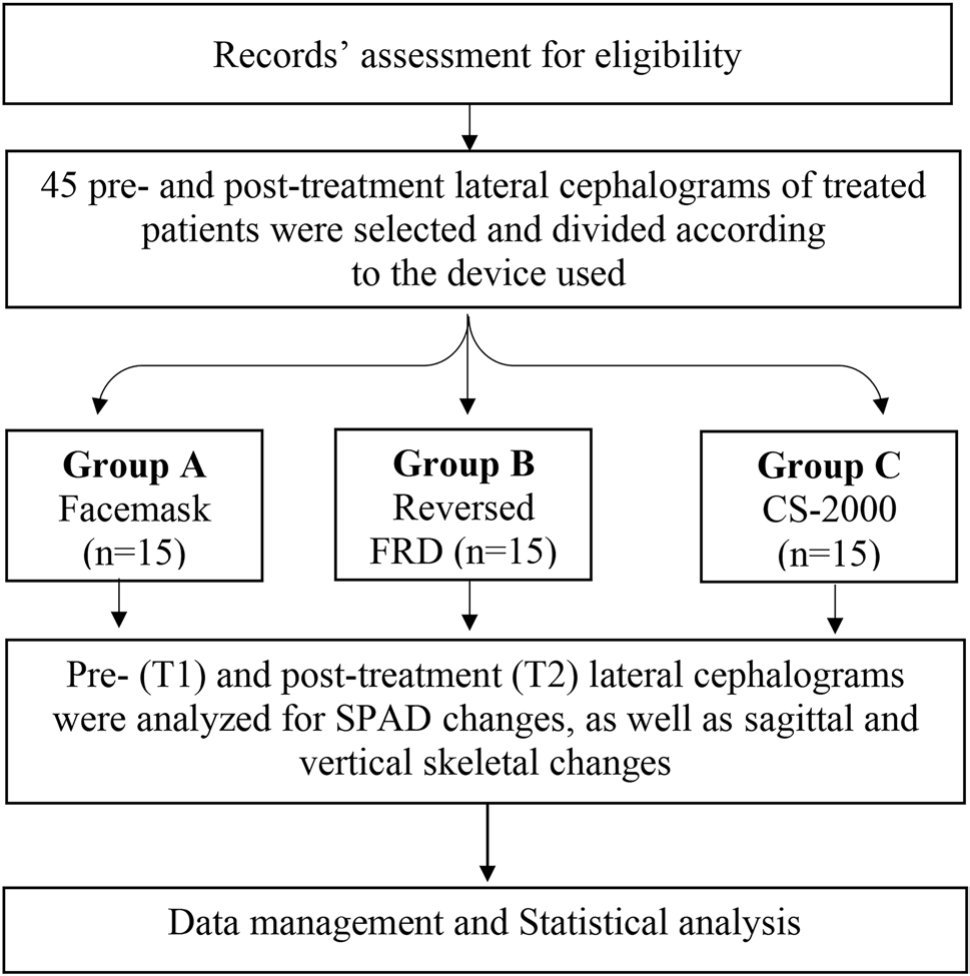
Research design flowchart.

The obtained lateral cephalometric x-rays were digitally traced using Dolphin Imaging software version 12.0 Premium (Dolphin Imaging, Chatsworth, CA, US), where various landmarks were identified. The sagittal pharyngeal airway space was divided into the Nasopharyngeal airway area (NPAA), the Oropharyngeal airway area (OPAA), and the Laryngopharyngeal airway area (LPAA)^28^. The upper border of NPAA was delineated by a line extending from the Harmonium (H) to the posterior nasal spine (PNS). The lower extent of the NPAA was traced by marking a line at the tip of the soft palate parallel to the Frankfurt Horizontal plane (FH), extending to the posterior wall of the pharynx. The OPAA and LPAA were differentiated by a line drawn at the level of the tip of epiglottis, parallel to the FH plane to the posterior wall of the pharynx. The lower border of the LPAA is defined as a line drawn parallel to FH plane, passing through the antero-inferior most point (C5AI) of the fifth cervical vertebra. The area has been measured using the same software in mm^2^ (Fig. 4). A research design flow chart summarizing the study procedures is presented in (Fig. 5).

### Sagittal and vertical skeletal measurements

From the obtained pre- and post-treatment lateral cephalograms and using the same software (Dolphin Imaging software version 12.0 Premium), several angular measurements were performed, where a sagittal as well as a vertical evaluation were carried out. For assessment in the sagittal dimension, the angles SNA, SNB, and ANB were measured. As for the vertical dimension, the Frankfurt-mandibular plane angle (FMA) was measured for evaluation.

### Blinding

The researcher was blinded during assessment of the obtained lateral cephalograms, and the statistician was also blinded throughout the data assessment process.

### Intra-examiner and Inter-examiner reliability

Initially, one researcher performed all the measurements. The same and another calibrated independent investigator repeated the whole measurements on 10 randomly selected x-rays, 2 weeks later to test intra and inter-examiner reliability using intraclass correlation coefficient (ICC)^29^. The calculated ICC ranged from 0.82 to 0.96 indicating good to excellent agreement between examiners and across time.

### Statistical analysis

Normality was checked for all variables using descriptive statistics, plots (Q-Q plots and histogram), and normality tests. All variables showed normal distribution, so means and standard deviations (SD) were calculated, and parametric tests were used. Comparisons of different parameters between the three study groups were done using One-way ANOVA, followed by multiple pairwise comparisons using Bonferroni adjusted significance level. Comparisons of different parameters between T1 and T2 within each group were done using paired samples t-test. Significance was set at *p* value < 0.05. Data were analyzed using IBM SPSS for Windows (Version 26.0).

## Results

Baseline demographic data regarding the patients included in each of the three study groups, with their respective treatment durations are represented in Table 1, where insignificant differences have been reported between them (*p* > 0.05). The mean and standard deviation for each of the measured variables were calculated at the onset (T1) and at the end of treatment (T2), as well as the differences between them (T2–T1), and are presented in Tables 2, 3.

**Table 1:** Baseline characteristics of the study sample.

		FM (n = 15)	Reversed FRD (n = 15)	CS (n = 15)	*p* value
Age	Mean (SD)	9.20 (0.66)	9.39 (0.53)	9.33 (0.40)	0.63
Gender: n (%)	Males	10 (66.7%)	7 (46.7%)	5 (33.3%)	0.19
	Females	5 (33.3%)	8 (53.3%)	10 (66.7%)	
Treatment duration (months)	Mean (SD)	7.45 (0.86)	7.17 (1.09)	7.61 (0.76)	0.43

**Table 2:** Comparison of the sagittal pharyngeal airway measurements (mm^2^) between the three study groups.

	FM (n = 15)	Reversed FRD (n = 15)	CS (n = 15)	*p* value 1
	Mean (SD)			
NPAA				
T1	353.53 (28.95)	377.87 (40.22)	385.27 (40.50)	0.06
T2	413.60 (35.10)^a^	453.93 (40.97)^b^	459.13 (41.73)^b^	0.005*
Difference	60.07 (20.09)^a^	76.07 (13.99)^b^	73.87 (6.13)^b^	0.009*
*p* value 2	< 0.001*	< 0.001*	< 0.001*	
OPAA				
T1	176.07 (13.64)	182.40 (16.85)	191.13 (28.87)	0.15
T2	206.47 (12.76)^a^	299.20 (18.07)^c^	260.20 (30.55)^b^	< 0.001*
Difference	30.40 (8.00)^a^	116.80 (26.36)^c^	69.07 (13.93)^b^	< 0.001*
*p* value 2	< 0.001*	< 0.001*	< 0.001*	
LPAA				
T1	282.07 (46.19)	320.60 (56.85)	307.07 (51.08)	0.12
T2	309.13 (46.36)^a^	397.33 (44.60)^b^	361.00 (58.97)^b^	< 0.001*
Difference	27.07 (7.46)^a^	76.73 (20.44)^b^	69.47 (6.95)^b^	< 0.001*
*p* value 2	< 0.001*	< 0.001*	< 0.001*	

SD, Standard deviation; CI, Confidence interval; *p* value 1, One-way ANOVA; *p* value 2, Paired samples t-test.

*Statistically significant at *p* value < 0.05.

a, b, c: different letters denote statistically significant differences between groups using Bonferroni adjusted significance level.

**Table 3:** Comparison of the sagittal and vertical skeletal measurements’ changes between the three study groups.

	FM (n = 15)	Reversed FRD (n = 15)	CS (n = 15)	*p* value 1
	Mean (SD)			
SNA°				
T1	75.97 (1.72)	76.53 (1.96)	75.53 (2.23)	0.39
T2	77.80 (1.90)	78.80 (2.24)	77.20 (2.37)	0.14
Difference	1.83 (0.53)^ab^	2.27 (0.46)^b^	1.67 (0.49)^a^	0.005*
*p* value 2	< 0.001*	< 0.001*	< 0.001*	
SNB°				
T1	78.85 (1.60)	79.47 (2.36)	78.60 (2.61)	0.55
T2	78.13 (1.64)	78.33 (2.26)	77.73 (2.60)	0.75
Difference	− 0.71 (0.67)	− 1.13 (0.52)	− 0.87 (0.52)	0.14
*p* value 2	0.001*	< 0.001*	< 0.001*	
ANB°				
T1	− 2.87 (0.91)	− 2.93 (0.88)	− 3.07 (0.96)	0.84
T2	− 0.33 (0.08)^ab^	0.40 (0.09)^b^	− 0.13 (0.09)^a^	0.03*
Difference	2.54 (0.75)^a^	3.33 (0.82)^b^	2.60 (0.74)^a^	0.01*
*p* value 2	< 0.001*	< 0.001*	< 0.001*	
FMA°				
T1	27.85 (2.62)	28.53 (3.25)	27.87 (1.55)	0.71
T2	30.17 (2.57)	30.60 (2.56)	29.87 (1.30)	0.67
Difference	2.32 (0.97)	2.07 (1.16)	2.00 (0.85)	0.66
*p* value 2	< 0.001*	< 0.001*	< 0.001*	

SD, Standard deviation; CI, Confidence interval; *p* value 1, One-way ANOVA; *p* value 2, Paired samples t-test.

*Statistically significant at *p* value < 0.05.

a, b, c: different letters denote statistically significant differences between groups using Bonferroni adjusted significance level.

### Measurement of the sagittal pharyngeal airway dimension

Changes in the sagittal pharyngeal airway area at the three measured levels NPAA, OPAA, and LPAA are presented in Table 2. Firstly, at the nasopharyngeal level (NPAA), all the Class III corrective appliances elicited a statistically significant increase in the NPAA between T1 and T2 (*p* < 0.05). On comparing the three examined appliances, the FM resulted in a significantly less increase in the airway area at this level (60.07 ± 20.09 mm^2^) in comparison to both the Reversed FRD, and the CS (76.07 ± 13.99 mm^2^, and 73.87 ± 6.13 mm^2^, respectively). However, insignificant differences have been noted between the Reversed FRD and the CS (*p* > 0.05).

At the second level (OPAA), the FM, the Reversed FRD, as well as the CS resulted in a statistically significant increase at T2 in comparison to the initial airway area (T1) (*p* < 0.05). The comparisons between the tested appliances revealed that the Reversed FRD elicited the greatest OPAA increase of 116.80 ± 26.36 mm^2^, followed by the CS with an increase of 69.07 ± 13.93 mm^2^, and then finally followed by the FM where an increase of 30.40 ± 8.00 mm^2^ has been registered, with the differences between the three of them being statistically significant (*p* < 0.05).

**Figure 6:**
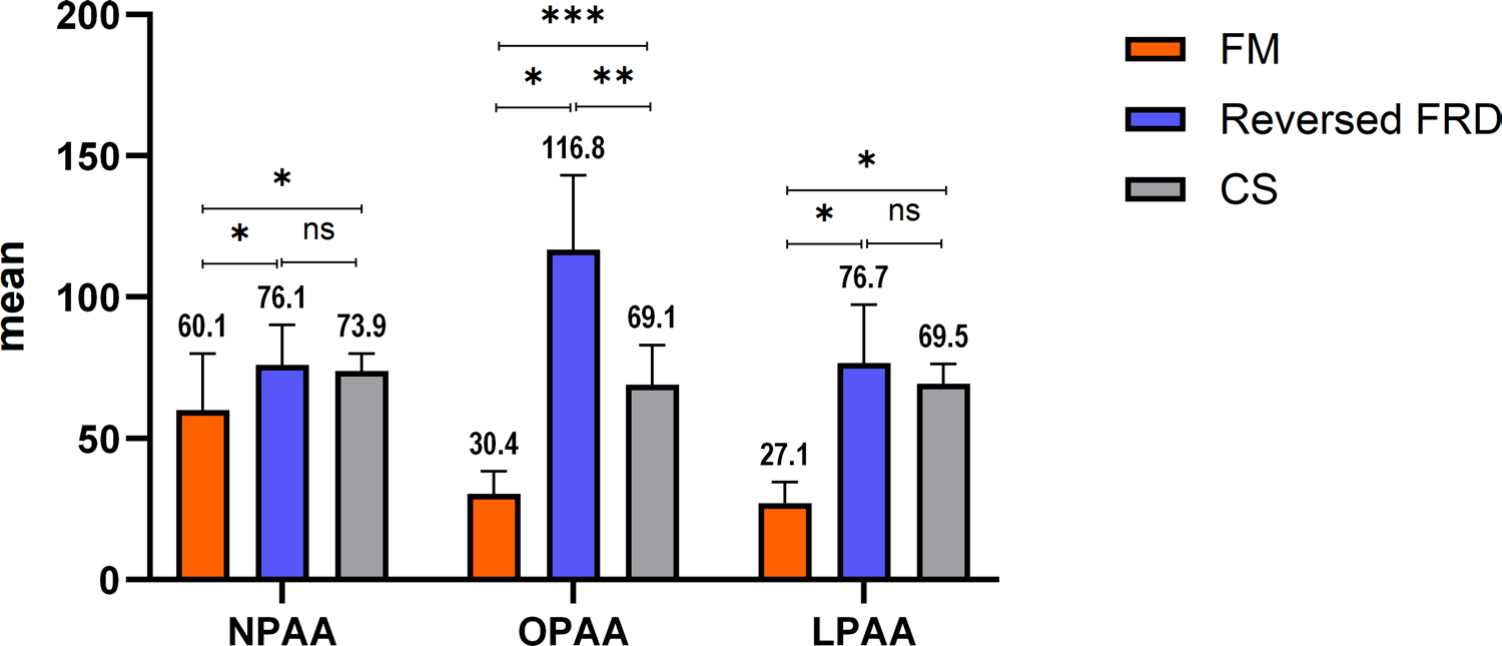
Changes in the sagittal pharyngeal airway area at NPAA, OPAA, and LPAA with the three investigated Class III corrective appliances.

At the final lowest pharyngeal level (LPAA), and similar to both NPAA and the OPAA, the investigated appliances brought about a significant increase at T2 in comparison to T1 (*p* < 0.05). However, in the FM group, a significantly less change has been observed (27.07 ± 7.46 mm^2^) in comparison to both the Reversed FRD and the CS (76.73 ± 20.44 mm^2^, and 69.47 ± 6.95 mm^2^, respectively). Nevertheless, on comparing the Reversed FRD and the CS, insignificant differences have been reported between both appliances (*p* > 0.05). Changes in the pharyngeal airway area at the three measured levels are represented in (Fig. 6).

### Sagittal and vertical skeletal measurements

**Figure 7:**
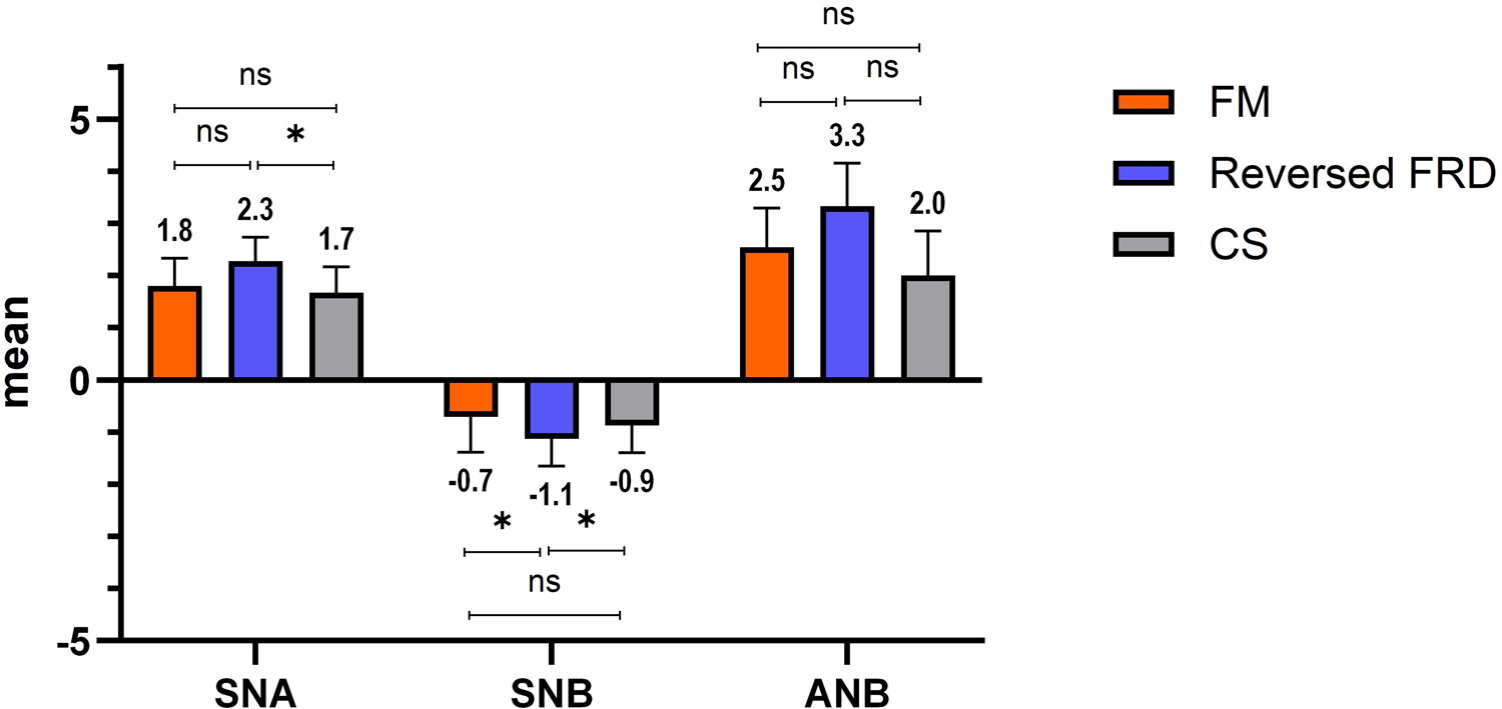
Sagittal skeletal changes in the three studied groups, pre-treatment and post-treatment.

Changes in both the sagittal and vertical dimensions in the three study groups, pre- and post-treatment, are presented in Table 3. Regarding the sagittal measurements, the FM, the Reversed FRD, as well as the CS elicited a significant increase in the SNA° values between T1 and T2 (1.83 ± 0.53 mm^2^, 2.27 ± 0.46°, and 1.67 ± 0.49°, respectively). No statistically significant difference has been shown between the FM group, in comparison to both the Reversed FRD and the CS (*p* > 0.05). However, SNA° values were significantly higher with the Reversed FRD in comparison to the CS (*p* < 0.05). As for the SNB angle, a significant decrease has been noted at T2 in comparison to T1 in all the tested groups, with a decrease of 0.71 ± 0.67° with the FM, and 1.13 ± 0.52° with the Reversed FRD, and finally a 0.87 ± 0.52° reduction with the CS, although no statistically significant differences have been noted between them (*p* > 0.05). Finally, for the ANB°, a statistically significant change has been observed between T1 and T2 in all groups. Insignificant ANB° differences were reported between the FM and CS groups (2.54 ± 0.75°, and 2.60 ± 0.74°, respectively), whereas a statistically significant change of 3.33 ± 0.82° was calculated in the Reversed FRD group in comparison to both the FM and the CS, representing the greatest ANB° change. The skeletal sagittal changes pre- and post-treatment using the three investigated Class III correctors are presented in (Fig. 7).

For the vertical dimension represented by the FMA° values, a statistically significant increase has been calculated at T2 when compared to T1 in the three tested groups, with the amount of change being 2.32 ± 0.97° with the FM, 2.07 ± 1.16° with the Reversed FRD, and 2.00 ± 0.85° with the CS, with no statistically significant differences between them (*p* > 0.05).

## Discussion

The current study was executed to assess the sagittal pharyngeal airway changes accompanying the use of three Class III correction appliances including the FM, the Reversed FRD, and the CS. According to the present outcomes, the null hypothesis has been rejected, where significant differences between the FM, the Reversed FRD, and the CS have been reported regarding the changes in the SPAD.

The age of the subjects chosen for this investigation ranged from 8 to 11 years (mean age 10.18 ± 0.75 years). It is to be noted that despite the fact that FM therapy has been advocated under the age of 8 years, since the impaired interdigitation of the intermaxillary suture at this young age will in turn favor an orthopedic maxillary reaction^30,31^, compliance of the treated patients at this age is usually rather questionable. Additionally, the difficulty in oral hygiene maintenance advocates the choice of an older age group.

The study design was retrospective, aiming to assess and compare between three Class III corrective appliances. No untreated control group has been included in the current investigation, as it is unethical to follow-up any type of developing malocclusion without intervening with an interceptive corrective action. Therefore, no records for a control group were available in the department’s archives for assessment. Retrospective studies evaluating pharyngeal airway changes that have not incorporated a control group include that of Oktay and Ulukaya^19^, in which the age range of the obtained sample was relatively close to that included in the current study records, therefore, a precedent has been established.

Transverse maxillary deficiency has been one of the exclusion criteria in the current study. This particular point was considered because of the impact of rapid palatal expansion (RPE) in conjunction with FM therapy on increasing the pharyngeal airway dimension^32^, due to the mobilization of the skeletal structures^12^. Therefore, to increase the veracity of our results, cases involving RPE have been excluded in order to assess the pure impact of the tested appliances on SPAD with no confounding factors, which also conforms to the guidelines followed by Husson et al.^33^.

Two-dimensional lateral cephalograms have been employed for the pre- and post-treatment assessment of SPAD, and they have also been the tools of choice for airway assessment by several authors^19,21,34,35^. Lateral cephalograms have been employed in the current study because of their diminished radiation dose, their cost-effectiveness, in addition to them being readily available as routine radiographs^28^. On another note, reproducibility of airway dimensions on lateral cephalograms has been proven to be of high accuracy^36^. Moreover, a high correlation has been reported between pharyngeal airway measurements on lateral cephalometric radiographs as well as those obtained through volumetric measurements performed on three-dimensional cone-beam computed tomography (CBCT)^37^.

Assessment of the recorded sagittal skeletal changes revealed a significant increase in the SNA°, a significant decrease in the SNB°, together with a significant increase in the ANB° at T2 in comparison with T1, with all the investigated Class III correctives. These outcomes have been previously explained with FM therapy as the result of prompting the sutural growth in the maxillary complex, by pulling the entire complex forward^18,38,39^. Additionally, this action brings about a clockwise downward backward rotation of the mandible or a redirection of the mandibular growth. The significant SNA° and SNB° changes consequently elucidate the significant change in the ANB° angle, which represents the intermaxillary relationship. The same explanation could also be relevant to the Reversed FRD and the CS. With the Reversed FRD, a pushing force is exerted on the mandible, whereas with the CS, a pulling force is exerted, both of which end up with a reciprocal force on the maxillary complex, guiding it into a forward direction. Moreover, it has been stated that the CS is an inter-arch spring-loaded module that acts full time on the maxillary sutures, which adds to the impact of the appliance^16^. Similar results have been observed with FM therapy by Oktay and Ulukuya^19^, and with the Reversed FRD by Eissa et al.^14^, except for the SNB° change which they reported to be insignificant, and this might be due to the fact that they have used a mini-screw supported Reversed FRD. As for the CS, relative results have been found by Vanlaecken et al.^16^.

On comparing the skeletal sagittal changes exerted by the FM, the Reversed FRD, as well as the CS, the greatest changes were designated to the Reversed FRD group in comparison with the rest, regarding the intermaxillary ANB angle. Accordingly, the employment of the Reversed FRD for the correction of Class III is advocated, with the additional advantage of being a non-compliance appliance, thus omitting the dependence on patients for successful results. The only study comparing between two Class III correction appliances was that by Yavan et al.^16^ comparing between FM and Reversed FRD. In their investigation, superior results were registered with FM use than with the Reversed FRD, which might be appertained to the use of RPE in conjunction with the FM.

For the vertical changes, a significant increase has been observed in FMA° at T2 in all treatment groups. Former research with FM therapy noted that the evident increase in the vertical face height is accredited in part to the clockwise rotation of the mandible, in addition to the probable downward forward movement of the maxillary complex after maxillary protraction^12^. However, with the Reversed FRD and the CS, the resultant clockwise mandibular rotation is the main contributory factor to the increased vertical face height, which is in accordance with Yavan et al.^12^, and Vanlaecken et al.^16^, respectively.

Regarding the SPAD, the FM, the Reversed FRD, and the CS elicited significant increases at the three measured levels (NPAA, OPAA, and LPAA) at T2 in comparison with T1. Airway changes accompanying FM therapy have been thoroughly studied in the literature, with the majority of the findings reporting a positive increase^19,21,22,33,40^, and a myriad of reasons were stated to account for this finding. One of the reasons was related to the protraction force of the FM which instigates forward maxillary movement, especially the PNS. In consequence, this might result in anterior displacement of the soft palate, which will eventually increase the upper airway dimension^20^. Another reason was related to the tongue position, which was reported to be altered by FM therapy. This observation could be prompted either by an increase in the volume of the oral cavity, or by the known clockwise mandibular rotation. The modified tongue posture could possibly result in an anterior repositioning of the soft palate, as well as an increase in the upper airway area^41^. As for the Reversed FRD and the CS, the significant increase in the SPAD could be attributed to the forward positioning of the maxilla in the sagittal plane, as well as the significant alteration in the intermaxillary relationship. Since no prior investigations have been conducted to test the influence of either appliance on the airway area, unfortunately, outcomes of the present study cannot be compared to others.

Comparisons between the SPAD changes at the three levels between the tested Class III correctives showed that the Reversed FRD elicited the greatest increase in the SPAD at the OPAA level, in comparison to both the FM and the CS. This reported superiority of the Reversed FRD could be appertained to its greater influence in the sagittal dimension (ANB° change) as reported earlier in the present study, suggesting a direct correlation between both the assessed outcomes. Thus, in addition to being a non-compliance appliance that is successful in improving a developing Class III condition, the Reversed FRD increases the SPAD significantly, thus overcoming any probable breathing problem in such patients.

Limitations of the current study include the absence of a long-term observation period after the antero-posterior correction, and the probable changes that might take place in the SPAD, which in turn justifies the need for further prospective clinical trials in this area. Also, the absence of a control group as a gold standard for comparison might be a limiting factor. Another limitation is the absence of data regarding patient compliance using FM therapy, since this is a major contributing factor to the success of treatment. Furthermore, the use of the two-dimensional lateral cephalometric radiographs is not the best method of assessment, in comparison to the three-dimensional methods which also allow volumetric airway evaluation.

## Conclusions

1. The FM, the Reversed FRD, and the CS, were found to generate a significant increase in the sagittal pharyngeal airway dimension, with the Reversed FRD contributing to the most significant change in the oropharyngeal airway area.
2. All the three appliances elicited significant sagittal skeletal improvements in the SNA°, SNB°, and ANB°, with the greatest intermaxillary change documented with the employment of the Reversed FRD.
3. The vertical skeletal dimension increased significantly with the investigated appliances, with the greatest change attributed to the FM.

## Data Availability

The datasets used and/or analyzed during the current study are available from the corresponding author on reasonable request.

## Abbreviations

MPA: Maxillary protraction appliance
FM: Facemask
FRD: Forsus fatigue resistant device
CS: CS-2000
NiTi: Nickel–titanium
SPAD: Sagittal pharyngeal airway dimension
CVMI: Cervical vertebral maturational index
NPAA: Nasopharyngeal airway area
OPAA: Oropharyngeal airway area
LPAA: Laryngopharyngeal airway area
H: Harmonium
PNS: Posterior nasal spine
FH: Frankfurt horizontal plane
C5AI: Antero-inferior point of the 5th cervical vertebra
FMA: Frankfurt-mandibular plane angle
ICC: Intraclass correlation coefficient
SD: Standard deviation
RPE: Rapid palatal expansion
CBCT: Cone-beam computed tomography
